# POOR SLEEP HEALTH MAY BUILD THE ONSET OF A FEAR OF FALLING AMONG COMMUNITY-DWELLING OLDER ADULTS

**DOI:** 10.1093/geroni/igad104.1831

**Published:** 2023-12-21

## Abstract

A greater fear of falling (FOF) predicts activity restrictions, falls, disability, and increases the risk of mortality among older adults. Although poor sleep has been identified as a relevant factor for FOF among older adults, evidence is primarily shown in cross-sectional studies using isolated sleep characteristics. Whether multidimensional sleep health contributes to increase in FOF among older adults who are particularly vulnerable to falls is less well understood. We investigated the longitudinal relationship between sleep health and the incidence of FOF among community-dwelling older adults and how the association differed between those with or without prior falls experiences. Data were from individuals who completed the sleep module in the National Health and Aging Trends Study (2013-2014; n=695). FOF was assessed with a single item. Multidimensional sleep health was measured with self-reported sleep items based on the SATED model (i.e., sleep satisfaction, daytime alertness, timing, efficiency, and duration). Covariates included sociodemographics, assistive device usage, health, and sleep medications. Multiple logistic regression was used to analyze the data. Poor sleep health was associated with the onset of FOF at 1-year follow-up (OR=1.20, 95%CI=1.02-1.41). Moreover, poor sleep health increased the odds of having FOF among individuals without prior falls experiences and elevated the already heightened risks of developing FOF among those who fell at baseline. Improving sleep health may potentially prevent older adults from developing FOF.

